# Mass Spectrometry-Based Spatial Imaging of the Cochlea

**DOI:** 10.1021/jasms.5c00436

**Published:** 2026-04-03

**Authors:** Roberto A. Ribas, Qun Tang, Scarlett I. Caffee, Cameron J. Shedlock, Tara R. Hawkinson, Abigail K. Dragich, Borhane E. C. Ziani, Franca Bucco Paolasso, Alyson P. Black, Xin Ma, Harshitha C. Kota, Li Chen, Gregory I. Frolenkov, Derek B. Allison, Matthew S. Gentry, Ramon C. Sun, Craig W. Vander Kooi

**Affiliations:** † Department of Biochemistry and Molecular Biology, 3463 University of Florida, Gainesville 32610, Florida, United States; ‡ Center for Advanced Spatial Biomolecule Research (CASBR), University of Florida, Gainesville 32610, Florida, United States; § Department of Physiology, 4530 University of Kentucky, Lexington 40536, Kentucky, United States; ∥ HTX Technologies, LLC, Chapel Hill 27516, North Carolina, United States; ⊥ Department of Biostatistics, College of Public Health and Health Professions & College of Medicine, University of Florida, Gainesville 32610, Florida, United States; # Department of Computer and Information Science & Engineering, University of Florida, Gainesville 32610, Florida, United States; ¶ Department of Otolaryngology - Head and Neck Surgery, University of Kentucky, Lexington 40536, Kentucky, United States; ∇ Markey Cancer Center and Department of Pathology & Laboratory Medicine, University of Kentucky, Lexington 40536, Kentucky, United States; ○ McKnight Brain Institute, University of Florida, Gainesville 32610, Florida, United States

## Abstract

Matrix-Assisted Laser Desorption/Ionization Mass Spectrometry Imaging (MALDI-MSI) is transforming spatial molecular studies. However, applying MALDI-MSI to small, anatomically complex tissues remains challenging. One such structure is the cochlea, the auditory part of the inner ear that is critical for hearing. To address these challenges, we developed and implemented a streamlined workflow for sample preparation and processing to obtain MALDI-MSI data on mouse cochlea. Sample acquisition was optimized to minimize time and processing steps, allowing use of flash-frozen neonatal mouse heads. This workflow enabled high spatial resolution metabolomic and lipidomic imaging of the sagittally cryosectioned mouse cochlea using *N*-(1-naphthyl) ethylenediamine dihydrochloride (NEDC) matrix via sublimation. Optimized NEDC sublimation allowed high signal-to-noise, reduced delocalization, and salt tolerance, allowing acquisition of 5 μm-resolution imaging data on a MALDI-MSI instrument. Sublimation was found to be superior to spraying as a method for matrix application due to its higher signal-to-noise, particularly for lipids and fatty acids, and improved spatial resolution. Diverse metabolites and lipids were measured throughout the cochlear region, revealing distinct spatial distributions. Clustering identified reproducible physiological regions, including the otic capsule and spiral ducts. High spatial resolution imaging revealed distinct tissues, cell types, and molecular signatures within the cochlea. These findings establish the utility of high spatial resolution MALDI-MSI for auditory research, enabling molecular mapping of cochlear function and dysfunction.

## Graphical abstract

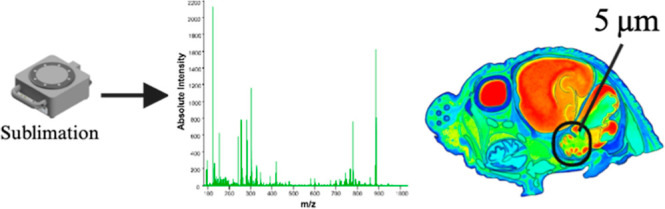

## Introduction

Matrix-Assisted Laser Desorption/Ionization Mass Spectrometry Imaging (MALDI-MSI), an emerging Mass Spectrometry (MS)-based technique that can detect the spatial distribution of metabolites, lipids, and other biomolecules, is transforming spatial molecular studies of diverse biological systems.^1–4^ MALDI-MSI is being applied to understand the fundamentals of dynamic biological processes, perturbations in disease states, and the efficacy of therapeutic interventions.^5–10^ Recent insights focusing on neuronal systems have begun to reveal the diverse metabolomic signatures and dynamic molecular processes which underlie key neuronal and sensory pathways.^4,11^ Recently, sensory systems have become the focus of investigation using MALDI-MSI, including the auditory system.

The auditory portion of the inner ear, known as the cochlea, is a complex spiral structure that converts mechanical inputs into electrical signals. Sound-induced fluid movement in the cochlea activates specialized sensory hair cells that transmit signals to spiral ganglion neurons (reviewed in^12^). Disruptions in this signal transduction pathway are a central cause of genetically inherited, environmental, and age-related hearing loss. Indeed, hearing loss is estimated to affect 2.5 billion people over the next two decades, with an annual global cost of ∼$1 trillion USD (World Health Organization, https://www.who.int/news-room/fact-sheets/detail/deafness-and-hearing-loss).

The cochlea represents a challenging application for MALDI-MSI. The cochlea is small, delicate, encapsulated deep in bone, and very heterogeneous in its cellular composition. While anatomically defined, the different cell types in the cochlea have highly differentiated roles, and the spatial dynamics of metabolites and lipids essential for cochlear metabolism and function are not well understood. Continuous sound exposure over a wide range of intensities may require dynamic metabolic adaptation within the cochlea. Bulk metabolomic studies of the cochlea have previously revealed how small molecule metabolites centrally integrate in cochlear function.^13^ Additional recent studies have begun to define key cochlear metabolites and how significant metabolic alterations are linked with noise-induced hearing loss.^14,15^ Most recently, metabolomic signatures of age-related hearing loss have been reported.^16^ However, metabolomic studies have been limited to pooled samples, leaving critical spatial cochlear metabolic information unexplored.

Initial applications of MALDI-MSI to the auditory system reported mapping of the distribution of five specific lipids in the cochlea.^17^ Subsequent studies mapped proteomic changes in noise-exposed mouse cochlea, revealing significantly altered proteins in specific, spatially distinct regions.^18^ Although MALDI-MSI can provide critical spatial molecular data, until recently it has been limited to tissue-level resolution (∼50 μm). Newly introduced options now exist that enable near single-cell resolution of ∼5 μm, which is essential for obtaining advanced biological insights.^19^

Recent developments that are advancing MALDI-MSI technology toward single-cell resolution include sample preparation methods, mass spectrometry hardware, and computational analysis.^20–23^ For sample preparation, matrix sublimation has emerged as a preferred method for high spatial resolution studies.^24^ MS hardware developments have focused on improvements in integral MALDI components leading to increased overall sensitivity, as signal-to-noise losses are typical when collecting at higher resolution. Finally, novel innovations in data analysis are extending the capabilities of MALDI-MSI analysis, enabling large scale and multiomics applications.^11^

Here, we describe the development and implementation of cutting-edge methodologies to provide spatial metabolomic and lipidomic MALDI-MSI data on the mouse cochlea. These data provide key insights into the spatial distribution of different classes of biomolecules in the cochlea and surrounding tissue, providing unique insights into the structure and function of distinct tissue and cell types in the auditory system.

## Experimental Section

### Mouse Models and Sample Preparation

Wild-type C57BL/6J mice were obtained from Jackson Laboratory. The day of birth was designated as postnatal day 0 (P0), with samples harvested at P2. Pups were euthanized by decapitation, and the heads were immediately frozen by placement directly above liquid nitrogen for 10 min. Frozen samples were then stored at – 80 °C until use. All animal procedures were approved by the University of Florida Institutional Animal Care and Use Committee under protocol number IACUC202200000541.

Whole heads were sectioned in the sagittal plane using a cryostat maintained at −18 °C. A Leica CM1860 cryostat (Leica Biosystems, Nussloch, Germany) was utilized to obtain sections at 10 μm thickness. Samples were mounted laterally onto a frozen Leica chuck using M-1 Embedding Matrix (Epredia, Portsmouth, NH, USA) as an adhesive medium for sectioning. Prior to sectioning, the opposite lateral side was trimmed on the cryostat to remove the skin and external ear, exposing the underlying structures until the cochlear region was fully visible. Sections were mounted onto positively charged microscope slides (71873-02, Electron Microscopy Sciences, Morgantown, PA, USA), dehydrated in a vacuum desiccator, and stored at −80 °C until further processed.

### Sample Preparation

Prior to matrix sublimation or spray, slides were removed from the −80 °C freezer and immediately placed in a vacuum desiccator for at least 1 h to equilibrate to room temperature. Slides were sequentially processed with *N*-(1-naphthyl) ethylenediamine dihydrochloride (NEDC) as a matrix to acquire full-spectrum, untargeted metabolomics and lipidomics. NEDC was applied via an HTX SubliMATE or HTX M5 Sprayer (HTX Technologies, Chapel Hill, NC, USA) using sublimation or spraying, respectively.

For sublimation, NEDC was resuspended at 13.3 mg/mL in 100% methanol, and 1.5 mL was used for each run (20 mg in 1.5 mL). The matrix solution was freshly prepared and sonicated for 10 min using alternating cycles of 30 s on and 30 s off. A 1.5 mL aliquot of matrix was dispensed into the SubliMATE wafer and allowed to evaporate for 1 min prior to sealing the SubliMATE chamber. The system was connected to a Labconco vacuum pump (model 117, Labconco, Kansas City, MO, USA). Following the evaporation step, the samples were secured to the inner lid of the chamber, positioned above the wafer, and vacuum was applied for 5 min with a metal container of dry ice/acetone placed on top. Samples were cooled to approximately −68 °C via the dry ice slurry on the cooling top, and the temperature monitored throughout. Vacuum pressure was equilibrated to <40 mTorr. After 5 min, the wafer was heated to 165 °C for 40 min to initiate sublimation. At the end of this 40 min, system pressure reached 1 mTorr. The wafer temperature was returned to room temperature, and the bucket of dry ice was replaced with prewarmed heat-sink and allowed 5 min to return to approximate room temperature (18–24 °C) to prevent condensation build-up during vacuum release. After a 5 min equilibration period, vacuum was released. Slides were recrystallized immediately following sublimation using 10% methanol. Slides were first secured on the inside of a Petri dish lid and placed directly on the bottom of a 50 °C oven, and preheated for 90 s with tissue facing up. After 90 s, the Petri dish lid was quickly flipped over onto the Petri dish itself, which contained Whatman’s filter paper saturated with 1 mL 10% methanol (tissue facing the filter paper), and returned to the 50 °C oven for another 90 s. Following this, the Petri dish containing the filter paper was rapidly removed, and the Petri dish lid containing the slides was left in the 50 °C oven for another 90 s, with tissue facing the bottom of the oven. Slides were then removed from the oven for drying.

For spraying, previously optimized methods were utilized.^4,25^ NEDC matrix solution was prepared at 7 mg/mL in 70:30 (v/v) HPLC-grade methanol/water. The solution was sonicated in an ultrasonic bath for 10 min at room temperature and filtered through a 0.22 μm PTFE syringe filter to remove undissolved particles. The filtered matrix solution was then deposited by automated pneumatic spraying using an HTX M5 Sprayer. Spraying parameters were as follows: 14 passes, flow rate of 0.06 mL/min, CC pattern, track spacing of 3 mm, nozzle velocity of 1200 mm/min, nozzle temperature of 30 °C, nitrogen pressure of 10 psi, and heated drying tray maintained at 50 °C.

### Data Collection and Processing

MALDI-MSI experiments were performed on a Bruker timsTOF fleX instrument (Bruker Scientific, Billerica, MA, USA). Prior to imaging, sodium formate was used for electrospray mass calibration, with the calibration mode set to high-precision calibration and zoom enabled at 0.01%. Imaging was conducted using a two-pass acquisition strategy, in which the cochlear region was first acquired at 5 μm spatial resolution using the microGRID, followed by a whole-section scan at 50 μm to capture the overall tissue profile. For 5 μm imaging, the laser application was set to custom mode with beam scanning disabled, resulting in a field size of 5 μm × 5 μm. Data were acquired using a single burst of 30 laser shots at a repetition rate of 10,000 Hz. For 50 μm imaging, beam scanning was enabled with a scan range of 46 μm × 46 μm, producing a 50 μm × 50 μm field size. Data were acquired using a single burst of 396 laser shots at 10,000 Hz. All measurements were acquired in negative ion mode using MS1 scan mode over a mass-to-charge ratio (*m*/*z*) range of 80–1500, with the following instrument settings: laser power 70%, MALDI plate offset 30 V, deflection 1 delta −70 V, Funnel 1 RF 150 Vpp, Funnel 2 RF 250 Vpp, multipole RF 250 Vpp, collision energy 10 eV, and collision RF 550 Vpp.

Postacquisition data were imported using SCiLS Lab 2026a (Bruker) and total ion count (TIC) normalized. Full feature annotation employed a hierarchical approach. Metaboscape (Bruker) was utilized, with the T-ReX 2D algorithm applied with intensity threshold and speckle width filtering. Ion deconvolution targeting [M-H]^−^, [M + Cl]^−^, and [M-H_2_O]^−^ ions was utilized as well as established libraries from in-house target lists,^4^ the latest HMDB Metabolite Library, MoNA, MassBank, FiehnLib, LipidBlast library, MetaboBASE Personal Library 2023, the Bruker Sumner MetaboBASE, and NIST 2022 libraries. Additional feature annotations were obtained from Metaboanalyst^26^ and Metaspace.^27^ Annotation accuracy was ensured through stringent *m*/*z* tolerances (5–10 ppm), mSigma values (25–250), and comprehensive parallel library searches, with unannotated features explored via SmartFormula for molecular formula prediction.

For further feature validation, ion mobility-derived collision cross section was used as an additional measure for identification as previously reported.^28^ MALDI imaging experiments were again conducted on the timsTOF fleX instrument, but operated in timsON mode under full-scan conditions without precursor isolation. The mass range was set to *m*/*z* 50–1100 Da using acquisition method (Table 1). Ion mobility separation was performed over a mobility range of 0.60–1.80 1/K_0_, with a ramp start voltage of 375.84 V, ramp end voltage of 164.38 V, and 917 ramp cycles, using a transit time of 3.6 ms. External mass and mobility calibration were carried out using the Bruker tuning mix, resulting in a TOF mass accuracy of approximately 0.19 ppm and an IMS calibration score greater than 0.999. Candidate ions were selected from the full timsON data set based on: (i) accurate mass within the *m*/*z* 50–1100 range, (ii) ion mobility separation for Collision Cross Section (CCS)-resolved features, and (iii) spatial localization within anatomically relevant cochlear regions (see Figures 2,4,5). CCS values were validated using established values,^29^ or based on values from Metaboscape (Bruker). Following completion of the timsON acquisition, data were processed using SCiLS Lab software applying the T-Rex2 algorithm with 55% coverage and an interval width of 2 Å. For each targeted *m*/*z* value, the corresponding CCS, derived from its mobility window, was assigned enabling export of the precise mobility and *m*/*z* parameters required for subsequent acquisition.

**Table 1:** Consolidated timsON Full-Scan and MS/MS Parameters

Category	Parameter	Value
Mass Range	*m*/*z*	50–1100 Da
Laser	20 μm	75% power
TIMS	mobility range	0.60–1.80 1/K_0_
	ramp start	375.84 V
	ramp end	164.38 V
	ramp cycles	917
	transit time	3.6 ms
	funnel 1 pressure	∼2.43 mbar
TOF	flight tube voltage	9900 V
	reflector voltage	2150 V
	detector TOF	2234.4 V
	pulser push/pull	1585.7 V
	digitizer interval	0.2 ns
	noise threshold	7
Calibration	TOF2 StdDev	0.000345
	mass accuracy	∼0.19 ppm
	IMS calibration score	>0.999
Collision Cell (MS/MS)	collision gas supply	50
	collision bias	25.5 V
	collision in/out	70 V/25.5 V
	collision RF	1100 Vpp
	collision RF MSMS	550 Vpp
iPRM Isolation	isolation *m*/*z*	targeted per compound
	1/*K*_0_ windows	from mobility
Spectral Processing	spectra summation	enabled (manual)
	software	dataAnalysis & metaboScape

**Figure 1:**
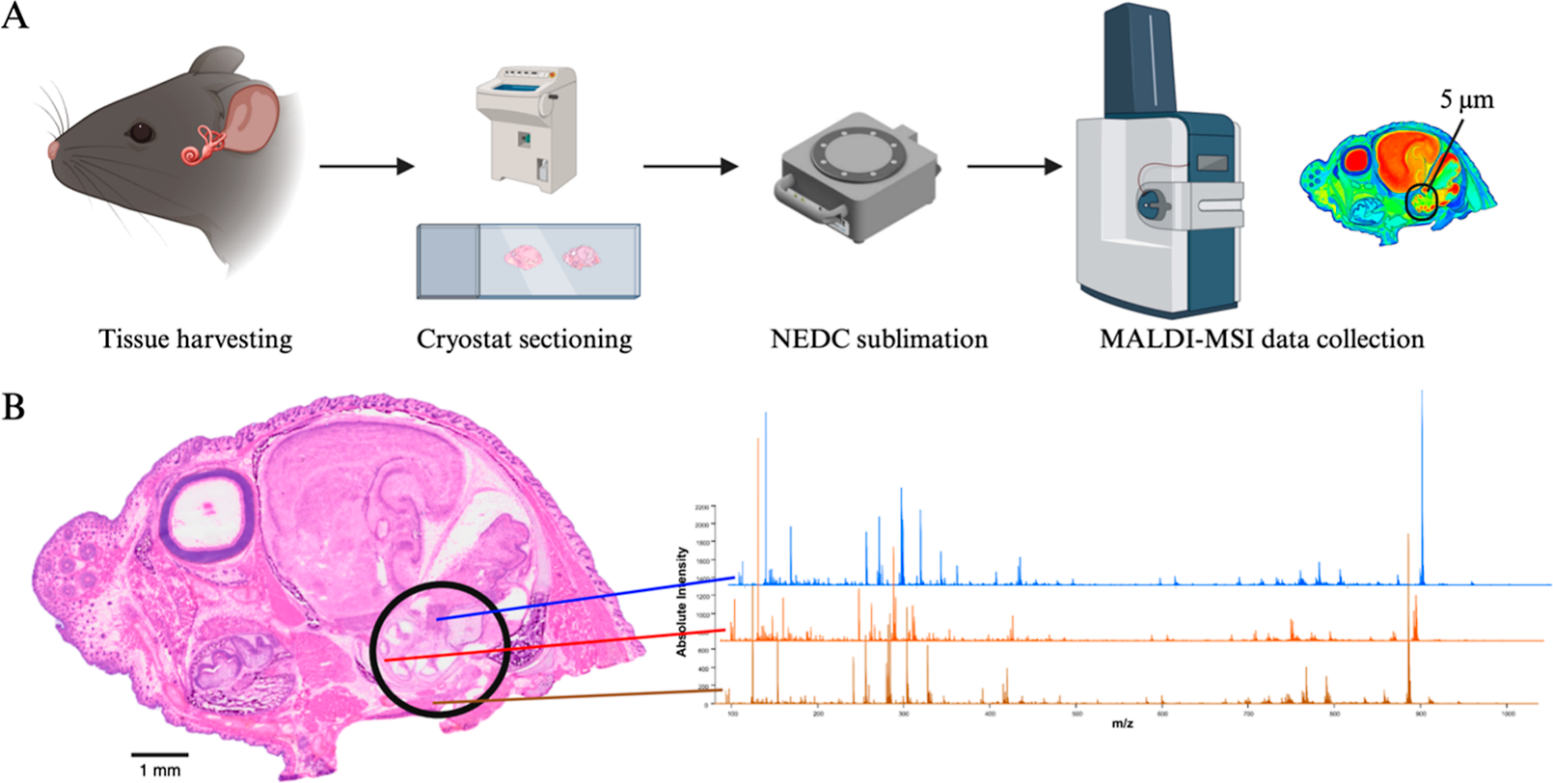
(A) Flow scheme for acquisition, preparation, and MALDI-MSI data collection of mouse cochlea. (B) H&E stain of the sagittal cut neonate head sample, with the cochlea indicated by the black circle. A stacked spectral plot of representative spectra from the auditory nerve, otic capsule, and muscle shows the diversity and range of metabolites and lipids measured. H&E staining was performed after MALDI-MSI data collection.

**Figure 2:**
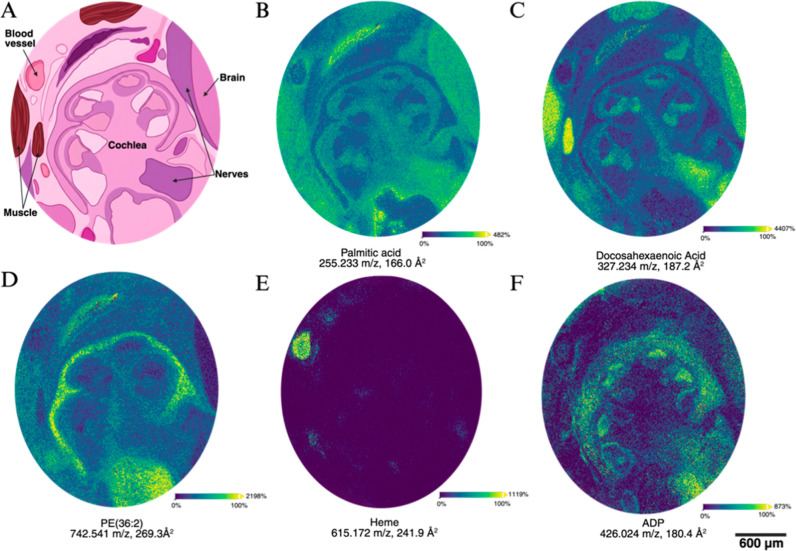
MALDI-MSI of the cochlea. (A) Schematic of the cochlea sample, with key anatomical regions labeled. (B–F) Select ion images of the spatial distribution of representative metabolites and lipids from NEDC sublimated samples, with corresponding *m*/*z* and CCS indicated below, demonstrating the range of distributions from broad, to specific, to cochlea-enriched. ADP, adenosine diphosphate.

**Figure 3:**
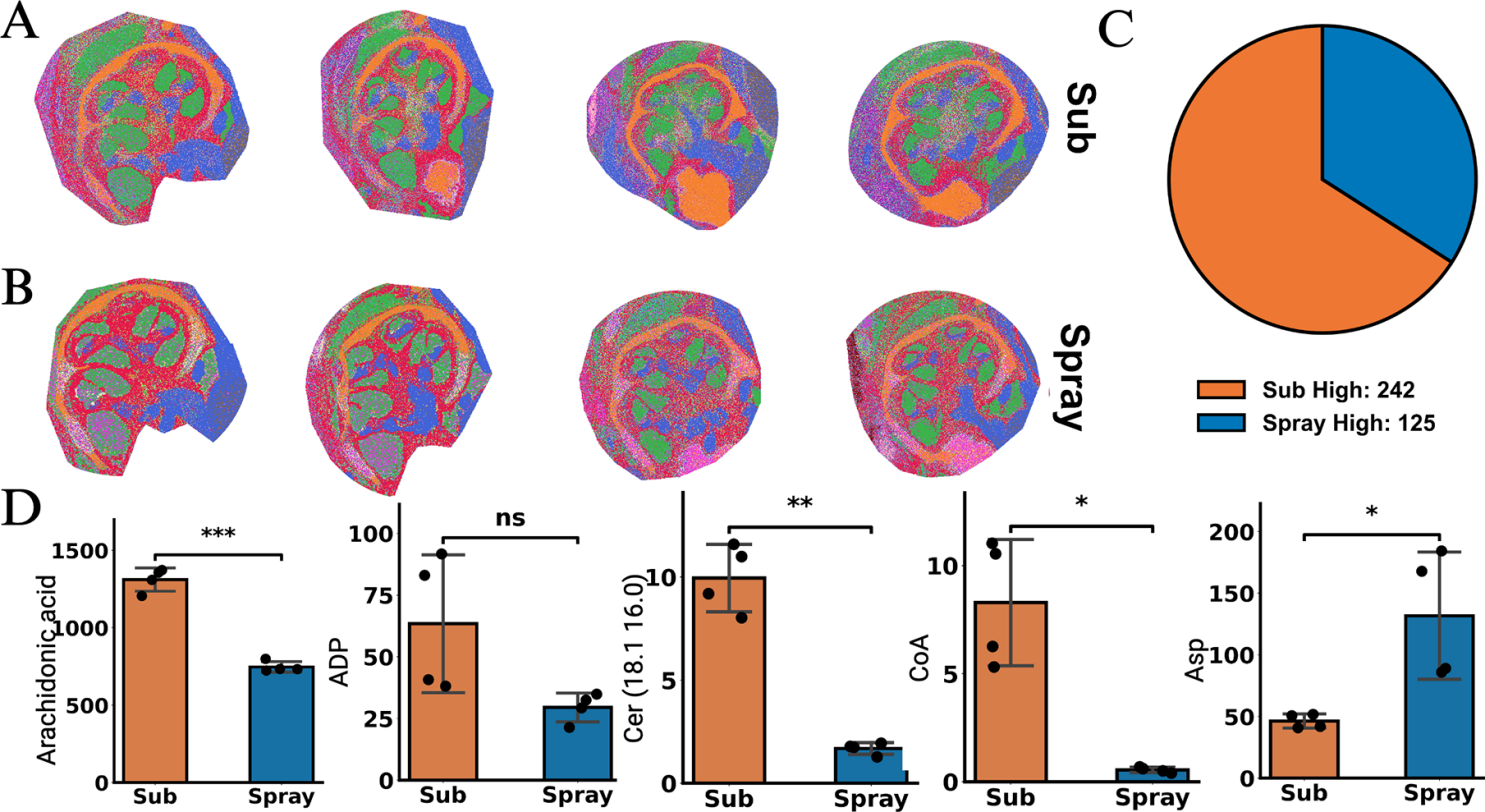
Clustering analysis of cochlea samples. Clusters from (A) sublimated and (B) sprayed cochlea from two paired biological and two technical replicates. (C) Comparison of signal intensities of sublimation and spray for all features. (D) Comparison of ion intensities of representative metabolites and lipids. Each graph shows the average and standard deviation, with individual data points displayed. *p < 0.05, **p < 0.01, ***p < 0.001, ns = not significant. Cer, ceramide; CoA, coenzyme A.

**Figure 4:**
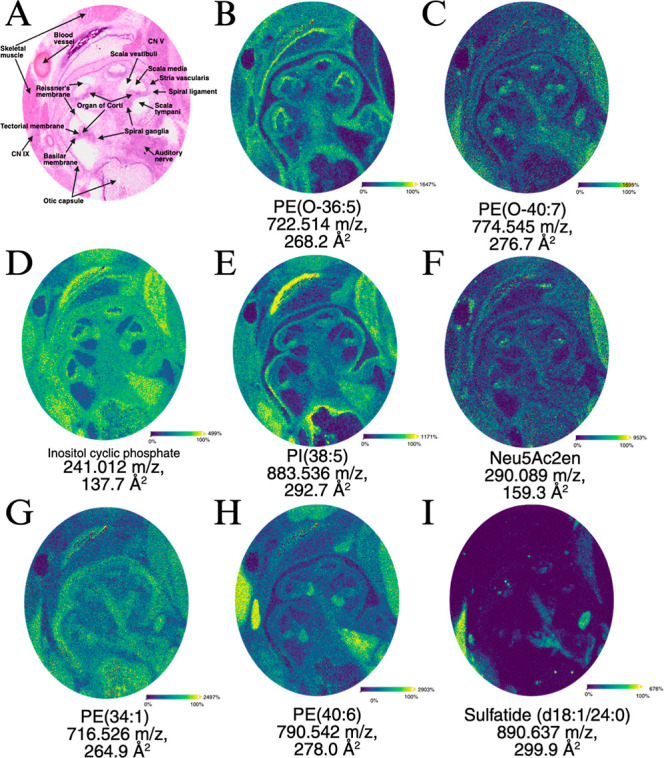
Molecular ions with unique cochlear localization. (A) H&E stain of the cochlear region, with key anatomical regions labeled. (B–I) Ion images of the spatial distribution of representative metabolites and lipids from NEDC sublimated samples, with corresponding *m*/*z* and CCS indicated below.

**Figure 5:**
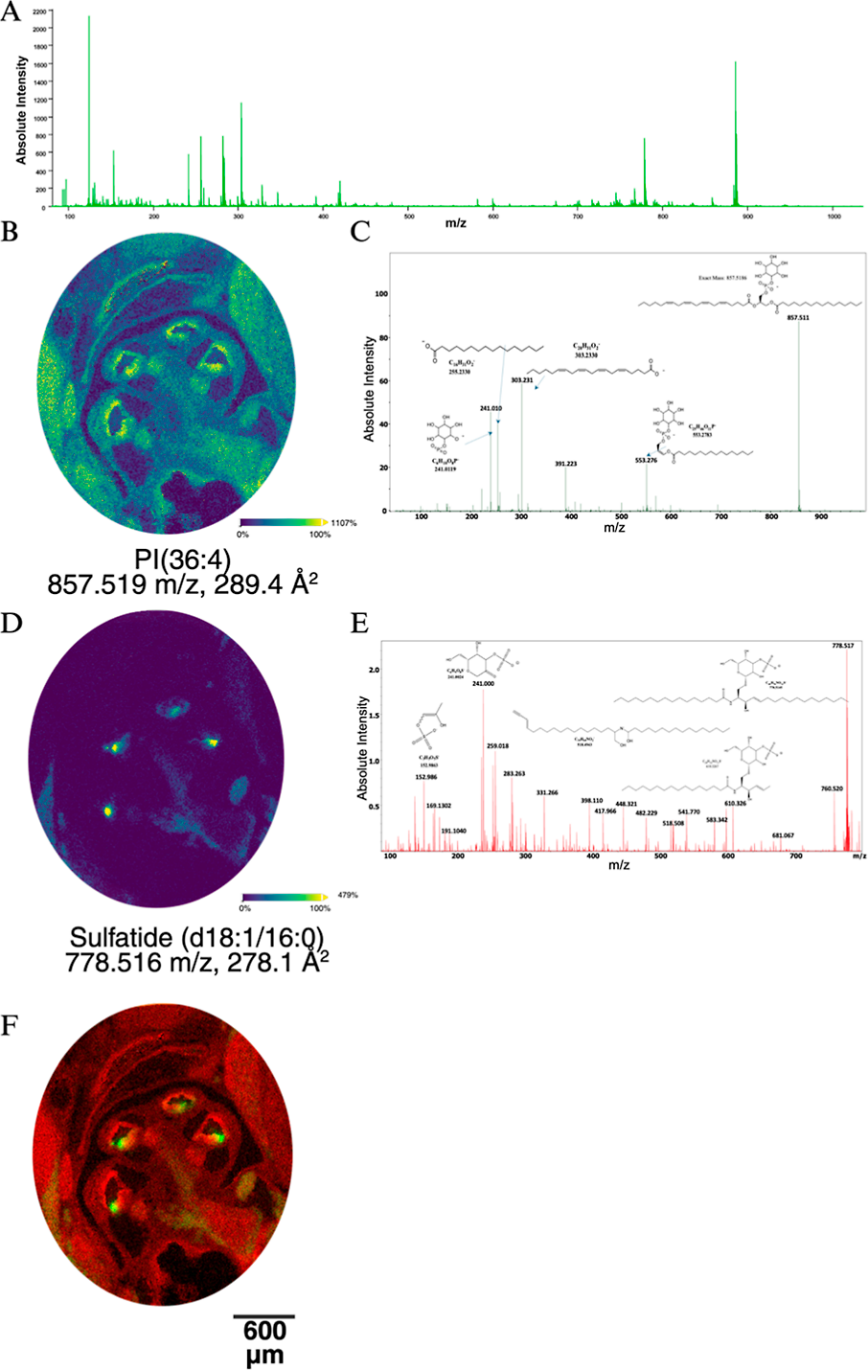
Molecular ions with unique organ of Corti localization. (A) MS of the local region of the organ of Corti. (B&D) Ion images of the spatial distribution of PI(36:4) and sulfatide (d18:1/16:0), respectively, with corresponding *m*/*z* and CCS indicated below. (C&E) MS/MS spectrum of PI(36:4) and sulfatide (d18:1/16:0), respectively, confirming the specific identity through diagnostic fragment ions produced from the selected precursor ion. (F) Direct superimposition of the PI from panel B pseudocolored red and the sulfatide from panel D pseudocolored green.

For each molecule, the precise mobility window (1/*K*_0_ start and end) was exported from the timsON data set and implemented in an iPRM method, enabling selective mobility filtering prior to fragmentation using specific mobility-resolved precursor windows. These mobility windows ensured isolation of structurally relevant conformers and minimized interference from isobaric or isomeric species prior to MS/MS acquisition. Collision-induced dissociation (CID) was performed using the instrument’s collision cell (collision gas supply 50; collision bias 25.5 V in negative mode), consistent with the global method to maintain spectral comparability. Regions exhibiting high abundance of each targeted ion in the cochlea were manually selected for specific features. Multiple spatial locations were interrogated for each precursor to confirm: reproducibility of fragmentation patterns, stability of precursor isolation, and absence of spatial artifacts. All MS/MS spectra corresponding to the same precursor were added to increase signal-to-noise ratio prior to annotation. MS/MS spectra were processed using Bruker DataAnalysis/Bruker MetaboScape. Fragment spectra were matched against internal and integrated MS/MS libraries. Annotation confidence was assessed based on fragment matching and MS/MS score. HMDB predicted MS/MS spectra were used for confirmation of observed fragments, Additionally, ChemDraw (Revvity, Waltham, Massachusetts, U.S.) fragmentation prediction tools were used to assess cleavage pathways.

All MALDI-MSI data reported in this study were exported in an imzML format and are fully available at https://sunlabresources.rc.ufl.edu MALDI_Cochlea.

### H&E Histology Staining

After MALDI data collection, tissue sections were stained with hematoxylin and eosin (H&E) histology stain in order to assist with anatomical annotation.^30^ Slides were immersed in 10% neutral buffered formalin for 30 min for fixation. Afterward, slides were rehydrated by immersion in 70% ethanol for 1 min and then deionized water for 3 min twice, using fresh water each time. A Leica ST4020 automated linear stainer was used in the staining process. Slides were dipped three times sequentially in each solution, after which slides were removed and then the process was completed two additional times before the slides were moved to the next solution. The following sequence of solutions was used: three hematoxylin baths, running water, two differentiation baths, running water, bluing solution, deionized water, 100% ethanol, eosin, two additional 100% ethanol baths. Lastly, slides were placed in xylene for clearing. Stained slides were covered with 24 × 50 mm coverslips at 0.13–0.17 mm thickness using UN1294 toluene solution as a mounting medium. Slides were allowed to dry overnight and then scanned using an Olympus VS200 slide scanner at 40× magnification.

### MALDI-MSI Alignment with Histology

To align the MALDI-MSI data with the corresponding H&E image, the first principal component (PC1) of the MALDI intensities through all metabolites was utilized as a fixed image. This provided a smooth and anatomically informative representation with strong contrast. The H&E image was resized to match the spatial dimensions of the MALDI image and treated as the moving image. Image registration was then performed using the SyN^31^ diffeomorphic transformation implemented in ANTsPy.^32^ Registration quality was evaluated using normalized mutual information and gradient correlation to quantify global alignment accuracy and structural consistency between the registered images.

### Cluster Analysis

A pixel-by-feature intensity matrix containing spatial coordinate metadata and annotations was exported using the SCiLS-Lab R API with TIC normalization. To optimize unsupervised clustering performance, feature intensities were log transformed and median absolute deviation (MAD) normalization was applied to feature intensities before clustering with clipping from −10 to +10 to minimize outlier effects. For clustering only, the MAD-normalized data were reduced to 50 principal components. Experimental variation between technical replicates was reduced to produce clusters that define distinct cochlear substructures based on feature signal profiles by performing Harmony^33^ (theta = 4.0, sigma = 0.2) on the principal component space to align samples while preserving biological heterogeneity. A k-nearest neighbor graph (*k* = 10) was constructed on this output followed by cluster detection. The Leiden algorithm at a resolution of 1.0 was used to assign each pixel to a distinct spatial cluster, resulting in 15 unique clusters.

Differential abundance analysis was performed to compare signal intensities between experimental groups of sublimation- and sprayer-applied matrix. The pixel intensities from the TIC-normalized SCiLS data frame were averaged across whole tissues or per discrete clusters. Statistical significance was assessed using Welch’s *t*-test. Data visualization was rendered programmatically using Python. Quantitative comparisons were visualized as bar charts representing group mean and standard deviation, where each individual replicate data point is shown. Statistical significance thresholds were annotated using *p < 0.05, **p < 0.01, and ***p < 0.001. Data were visualized using Prism 10 (GraphPad).

## Results and Discussion

### Preparation of NEDC Sublimated Sample

To obtain cochlear samples suitable for spatial metabolomic and lipidomic analysis, a rapid protocol minimizing sample handling was developed (Figure 1A). Neonatal mice were rapidly harvested by decapitation and the heads were frozen. The heads were then directly sectioned in a sagittal orientation using a cryostat. After sectioning and dehydration, slides were subjected to matrix application. To maximize the range of metabolites measurable and the spatial resolution, sublimation using NEDC was performed. NEDC is a preferred matrix for metabolomic studies due to its high signal-to-noise, low background, and salt tolerance.^34^ However, due to its propensity for decomposition and negligible vapor pressure, it has typically not been suitable for sublimation. With careful control of temperature, use of dry ice, extended sublimation time to achieve a low pressure, and recrystallization, sublimation of slides with NEDC was successfully optimized. This enabled MALDI-MSI data acquisition of diverse metabolites and lipids at 5 μm, for the region of interest surrounding the cochlea (Figure 1B).

### MALDI-MSI of Mouse Cochlea

The sagittal orientation of the samples allowed direct visualization of the key regions of the cochlea and surrounding tissue, which revealed the spatial molecular distribution of diverse metabolites and lipids. Data were collected at 5 μm for the cochlear region, based on anatomical landmarks (Figure 2A). Different ions highlighted key differences in the tissues and cellular types in the cochlea and surrounding regions (Figure 2B–F). Several metabolites and lipids were broadly distributed with some local enrichment, including fatty acids (Figure 2B,C). In contrast, other molecular species exhibited sharply restricted patterns across tissues and cell types. For example, one phosphatidylethanolamine (PE) species was enriched in the otic capsule (Figure 2D), illustrating the spatial fidelity of individual lipid signals at this scale. Further, blood vessels were clearly localized via the heme from intravascular blood (Figure 2E).

In addition to clear differences in tissues, a small number of molecules showed distribution differences between endolymph and perilymph, the key fluids associated with the spatially separated faces of the hair cells in the organ of Corti. For example, ADP was visualized at a robust signal-to-noise and appeared significantly higher in the endolymph compared to the perilymph (Figure 2F). The ability to measure and compare the composition of different biofluids is a unique advantage to this rapid and minimally disruptive sample preparation protocol combined with the sublimated NEDC matrix. This may enable future quantitative studies of the unique biofluids and tissues in the cochlea in normal hearing and in models of hearing loss.

Highly localized enrichment or depletion of multiple metabolites and lipids was apparent in different cochlear regions, discussed in detail below.

### Assessment of Reproducibility

Taken together, these results suggested that spatial molecular insights in highly localized regions of the cochlea are obtainable using this method. To establish that the method was rigorous and reproducible, data from two biological replicates (different animals) and two technical replicates (sequential slices) were collected, and spatial clustering analysis was applied to the data. Discrete spatial clusters, which directly align with physiological regions, were readily apparent (Figure 3A). In particular, the distinct cochlear morphology and organization were apparent within the otic capsule where the spiral ducts were reproducibly identified.

Additionally, these analyses allowed direct comparison of sublimation and spraying as matrix application methods. Clustering analysis showed clearly delineated physiological regions with both methods of matrix application, indicating significant continuity (Figure 3A,B). However, the overall resolution of the sublimated data was superior. Using edge sharpness assessment of the otic capsule boundary, intensity profiles from the sublimated sample indicated the spatial resolution was 5 μm, the resolution limit of the MALDI data collection. In contrast, intensity profiles from the sprayed sample indicated 10–15 μm spatial resolution, even with the same data collection parameters. Quantitative differences were also observed. As a whole, sublimation showed higher signal-to-noise for 66% of the molecular ions assigned (Figure 3C). The gain in signal was striking for certain molecules. Consistently, lipid and fatty acid species showed significant advantages with sublimation, as seen for representative species (Figure 3D). Other species showed no statistical difference between the two methods. In contrast, polar and charged metabolites, such as aspartate (Asp) (Figure 3D), showed higher signal-to-noise in the sprayed samples.

### Toward High-Resolution Cochlea MALDI-MSI

These data support the ongoing progress of the field to collect high-resolution MALDI-MSI data by improvements in sample preparation methods.^35^ Notably, the intensity of specific ions and their spatial distributions were highly reproducible, had high sensitivity, and high spatial resolution. These features allowed deeper insights into cochlear molecular distribution. Focusing on molecules with unique cochlear localization, significant signatures were observed. Co-registered histological staining of the sample after collection allowed identification and validation of specific regions of the tissue (Figure 4A). Noting the detailed organization of the cochlea provides landmarks for interpreting the data. An ether-linked PE (722.514 *m*/*z*) shows enrichment in the cochlear duct, which contains the endolymph (Figure 4B), while another ether-linked PE (774.545 *m*/*z*) is enriched in the basilar membrane (Figure 4C). Inositol cyclic phosphate (241.012 *m*/*z*) is found in the cochlea with particular enrichment in nerves (Figure 4D). A distinct phosphatidylinositol (PI) (883.536 *m*/*z*) shows similar basilar membrane localization as one of the PE lipids, but with greater distribution outside the cochlea (Figure 4E). The metabolite Neu5Ac2en (290.089 *m*/*z*) is selectively enriched in local regions of the cochlear duct centered in the organ of Corti, including the regions containing the tectorial membrane and hair cells (Figure 4F). Specific molecular species including polyunsaturated PE (790.542 *m*/*z*) define the nerves, highlighting the auditory nerve and spiral ganglia (Figure 4H). The class of molecules which showed the most distinct and focal localization were the 3-*O*-sulfogalactosylceramides, also known as sulfatides. This unique class has previously been extensively characterized by MS in samples from the mouse brain,^28^ and has been shown to be suitable for MALDI analysis.^36^ One sulfatide (890.637 *m*/*z*) showed highly restricted localization in nerves, as well as small specific foci (Figure 4I).

To assess the features that can be interrogated at this high spatial resolution, the organ of Corti was examined. It had a unique composition of metabolites and lipids (Figure 5A). A specific PI (857.519 *m*/*z*) is highly enriched in the periphery of the scala media, including the organ of Corti, the peripheral cells of the cochlear duct, and in the spiral ganglia (Figure 5B). MS/MS data were used to validate the molecular assignment (Figure 5C). A sulfatide (778.5162 *m*/*z*) is found to be highly localized in the organ of Corti (Figure 5D). Tandem mass spectrometry (MS/MS) data were used to validate the molecular assignment (Figure 5E). While the ion was lower intensity, this is a known mouse brain sulfatide^28^ and the MS/MS spectra show the hallmark peaks for the sulfatide headgroup.^36^ Superimposition of two of the molecular features that are enriched in the organ of Corti (Figure 5E) demonstrated the benefit of high spatial resolution. It is apparent that the sulfatide localization is distal to the tectorial membrane and represents a unique hallmark of critical cells in the organ of Corti.

Together, these data define critical features of the metabolic and lipidomic spatial biology of the cochlea. Precise analyte detection and overlay of histochemical and metabolic imaging indicate that the results can provide valuable spatial insight into cochlear function and represent a significant advancement toward high spatial resolution MALDI-MSI.

## Conclusions

This work reports the development and validation of a robust protocol for high-resolution MALDI-MSI for spatial molecular imaging of the mouse cochlea. A workflow including minimal sample handling, NEDC matrix sublimation, and matrix recrystallization was optimized to allow 5 μm-resolution spatial data to be reproducibly obtained. This approach enabled the untargeted mapping of diverse metabolites and lipids, revealing distinct spatial distributions across cochlear substructures. Specific lipids and metabolites had distinct localizations and enrichments, associated with the diverse and differentiated cellular architecture of the cochlea. In particular, unique lipid and sulfatide spatial distributions were defined.

These findings advance high spatial resolution MALDI-MSI for auditory research, offering molecular maps of the cochlea. These data highlight the potential of spatial metabolomics in elucidating the molecular underpinnings of auditory function, overcoming limitations of bulk analyses. Additionally, by preserving biofluid integrity, this method can provide novel insights into dynamic cochlear metabolism. Because mice begin hearing around postnatal day 14, this approach also enables spatial metabolomic studies during cochlear development and maturation of the auditory system. Further, it also can enable mechanistic comparisons across diverse models of hearing loss to identify convergent or divergent metabolic signatures. These include studying metabolic causes and contributions in genetic, noise, and drug-induced forms of hearing loss. Additionally, metabolic signatures may be useful in defining the efficacy of therapeutic interventions.

The current study has direct application for spatial metabolomic studies of other similar, small biological structures and those which benefit from single-cell or near single-cell resolution. Additionally, with the remarkable recent improvements in MALDI-MSI, there is significant potential for further advances. Adjusting MALDI sample preparation parameters was necessary for high quality images of these small structures, and future advances will continue to improve sample preparation, matrix application, spatial resolution, and signal-to-noise, allowing higher quality and more detailed studies of biological samples.

## Abbreviations

MALDI-MSI: matrix-assisted laser desorption/ionization mass spectrometry imaging
MS: mass spectrometry, NEDC, *N*-(1-naphthyl) ethylenediamine dihydrochloride
TIC: total ion count
H&E: hematoxylin and eosin
MAD: median absolute deviation
PE: phosphatidylethanolamine
PS: phosphatidylserine
PI: phosphatidylinositol
ADP: adenosine diphosphate
Cer: ceramide
CoA: coenzyme A
Asp: aspartate.
